# Neutrophil/lymphocyte ratio is a more sensitive systemic inflammatory response biomarker than platelet/lymphocyte ratio in the prognosis evaluation of unresectable pancreatic cancer

**DOI:** 10.18632/oncotarget.21340

**Published:** 2017-09-27

**Authors:** Yuan Gao, Wen-Jie Wang, Qiaoming Zhi, Meng Shen, Min Jiang, Xiaojie Bian, Fei-Ran Gong, Chong Zhou, Lian Lian, Meng-Yao Wu, Jun Feng, Min Tao, Wei Li

**Affiliations:** ^1^ Department of Oncology, The First Affiliated Hospital of Soochow University, Suzhou, China; ^2^ Department of General Surgery, The First Affiliated Hospital of Soochow University, Suzhou, China; ^3^ Department of Hematology, The First Affiliated Hospital of Soochow University, Suzhou, China; ^4^ Department of Radiation Oncology, Xuzhou Central Hospital, Xuzhou, China; ^5^ Department of Oncology, Suzhou Xiangcheng People's Hospital, Suzhou, China; ^6^ PREMED Key Laboratory for Precision Medicine, Soochow University, Suzhou, China; ^7^ Center for Systems Biology, Soochow University, Suzhou, China

**Keywords:** pancreatic cancer, systemic inflammatory response (SIR), neutrophil to lymphocyte ratio (NLR), platelet to lymphocyte ratio (PLR)

## Abstract

Multiple cancers arise from sites of infection, chronic irritation, and inflammation. It has been widely accepted that pancreatic cancer is an inflammation-driven cancer. In this study, we investigated the application value of systemic inflammatory markers, neutrophil to lymphocyte ratio (NLR) and platelet to lymphocyte ratio (PLR), in the prediction of chemotherapy response and prognosis in patients with late pancreatic cancer. 122 patients with inoperable pancreatic cancers were included and separated into two groups according to median values of NLR or PLR (NLR low:<3.81 or NLR high:≥3.81, and PLR low:<142.14 or PLR high≥142.14, respectively). Baseline NLR and PLR levels were significantly higher in pancreatic cancer patients compared with the healthy subjects. Neither of the baseline NLR or PLR levels could predict outcomes. Patients with low baseline level of NLR, but not PLR, had better responses to chemotherapy. Changes in NLR, but not PLR levels, were associated with the therapeutic efficacy. Patients who stayed in or dropped into the low NLR level subgroup after first-line chemotherapy had better responses, comparing to those stayed in or jumped into the high NLR level group. No similar results could be observed when the PLR level was investigated. Therefore, NLR is a more sensitive biomarker than PLR in the prediction of chemotherapy response of patients.

## INTRODUCTION

Pancreatic cancer is an extremely malignant solid cancer with a collective 1-year survival of just 26%, and 5-year survival less than 5%. Almost all of the patients have lost the chance of surgery at the time of diagnosis. Even for those who received surgery, they might finally suffer from recurrence or metastasis. Chemotherapy is still one of the major treatments for pancreatic cancer [1] [2] [3].

Systemic inflammatory response (SIR) is reported to be closely related to outcomes in many cancers, including pancreatic cancer [4]. It has been proved that neutrophil, lymphocyte and platelet levels in peripheral venous blood could be affected by tumor-induced SIR [5]. Therefore, the quantifications of these hematological parameters have been analyzed as markers of SIR in various malignant tumors, including pancreatic cancer [6, 7].

Researches have found that both platelet to lymphocyte ratio (PLR) and neutrophil to lymphocyte ratio (NLR) have predictive value in early diagnosis, and can reflect prognosis to some extend. It is reported that elevated levels of NLR usually suggested poor prognosis in patients with colorectal cancer who underwent primary resection or hepatectomy for liver metastases [6, 8]. PLR is regarded as a biomarker combining the apparent pre-inflammatory and prophylactic status of cancer with endogenous residual cancer resistance [9].

NLR and PLR have been proved to be both reliable and cost-effective [10], giving them promising prospects in the diagnosis and treatment of cancers. However, the sensitivities of NLR and PLR have rarely been compared in pancreatic cancer. In our study, we tried to evaluate the value of PLR and NLR in chemotherapy response and outcomes of patients with advanced pancreatic cancer.

## RESULTS

### Baseline levels of NL\R and PLR were higher in patients with pancreatic cancer comparing with the controls

**Figure 1:**
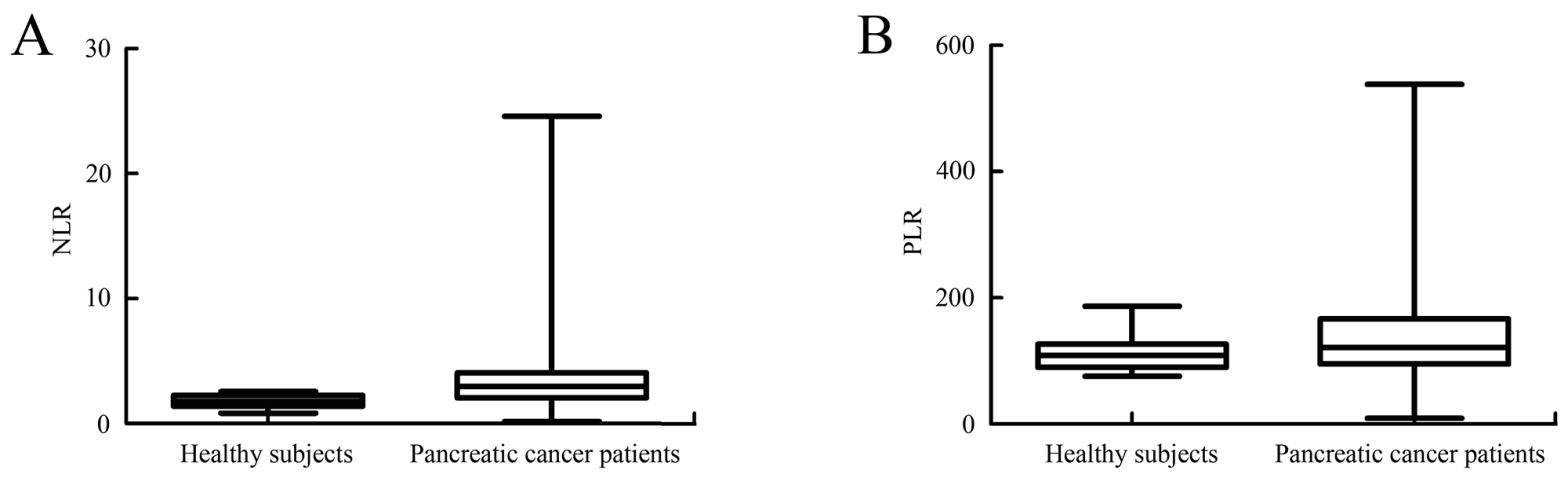
Comparation of baseline NLR and PLR values in pancreatic cancer group with the ones in healthy subjects **(A)** Comparation of baseline NLR values in pancreatic cancer group with the ones in healthy subjects. Baseline NLR level was significantly higher in pancreatic cancer patients than in the healthy subjects (*P*<0.001). **(B)** Comparation of baseline PLR values in pancreatic cancer group with the ones in healthy subjects. Baseline PLR level was significantly higher in pancreatic cancer patients comparing with the healthy subjects (*P*<0.001).

The mean baseline NLR level of pancreatic cancer patients was 3.81±3.65, and the corresponding level of healthy subjects was 1.80±0.48. Patients with pancreatic cancer had a significant higher baseline level of NLR than the controls (*P*<0.001) (Figure 1A). The mean baseline PLR level of pancreatic cancer patients was 142.14±86.76, while the corresponding level of healthy subjects was 112.34±25.52. Pancreatic cancer patients also had a higher baseline PLR level comparing with the controls (*P*<0.001) (Figure 1B). Increased baseline NLR and PLR values in pancreatic cancer group suggested that these biomarkers could be used to differentiate pancreatic cancer patients from healthy controls and might be used in the early diagnosis of pancreatic cancer.

As presented in Table 1, high baseline PLR and NLR levels both correlated with smoking history. High baseline level of PLR was related to liver metastasis as well.

**Table 1:** Relationship between baseline NLR, PLR level and clinicopathological features

Clinicopathologic features	n	NLR				PLR			
		Low (n)	High (n)	χ2	*P* value	Low (n)	High (n)	χ2	*P* value
Gender									
Men	74	40	34	1.788	0.181	34	40	0.787	0.375
Women	48	20	28			26	22		
Age (years)									
<65	58	26	32	0.838	0.360	28	30	0.036	0.849
≥65	64	34	30			32	32		
BMI (Body mass index)									
<25	60	28	32	0.298	0.585	29	31	0.034	0.854
≥25	62	32	30			31	31		
Smoke									
No	53	35	18	10.655	0.001**	32	21	4.701	0.030*
Yes	69	25	44			28	41		
Liver metastasis									
No	43	29	14	8.860	0.003**	20	23	0.189	0.664
Yes	79	31	48			40	39		

### Relationship between baseline NLR level and baseline PLR level

**Figure 2:**
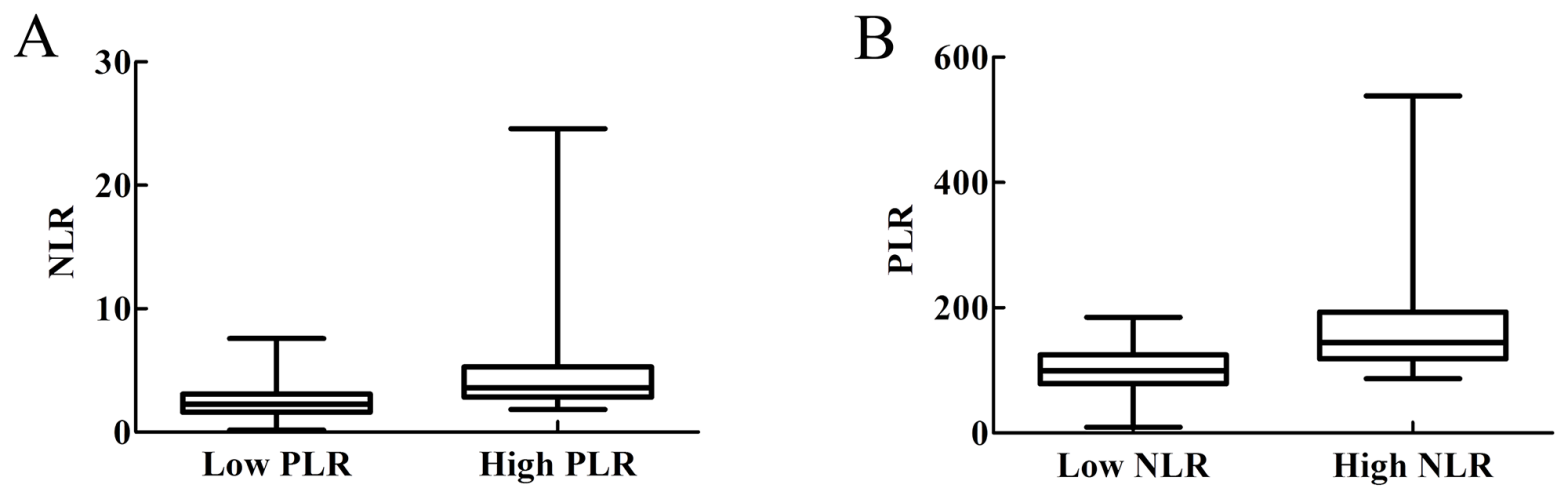
Relationship between baseline NLR level and baseline PLR level **(A)** Patients were divided into low and high PLR groups. NLR level of patients in high PLR group were higher than that of subjects in low PLR group (*P*<0.001). **(B)** Patients were divided into low and high NLR groups. PLR level of patients in high NLR group were higher than that of subjects in low NLR group (*P*<0.001).

To investigate whether baseline NLR level and baseline PLR level were associated, patients were divided in to low and high PLR or NLR goups, according to baseline PLR or NLR levels respectively. As shown in Figure 2A, NLR levels of patients in high PLR group were higher than those in low PLR group (*P*<0.001). Likewise, PLR levels of patients in high NLR group were higher than those in low NLR group (*P*<0.001, Figure 2B). Therefore, baseline NLR level and baseline PLR level correlated with each other.

### Baseline NLR level predicted chemotherapeutic efficacy

The relationships between baseline PLR or NLR levels and chemotherapeutic efficacy were presented in Table 2 and Table 3. The value of baseline NLR correlated with chemotherapeutic efficacy. However, baseline PLR level was not associated with chemotherapeutic efficacy.

**Table 2:** Relationship between NLR baseline levels and chemotherapeutic efficacy

NLR levels	PR+SD (n=76)	PD (n=46)	χ2	*P* value
Low (n=60)	47	13	12.929	<0.001
High (n=62)	29	33		

**PR: partial response; SD: stable disease; PD: progressive disease.**

**Table 3:** Relationship between PLR baseline levels and chemotherapeutic efficacy

PLR levels	PR+SD (n=76)	PD (n=46)	χ2	*P* value
Low (n=60)	33	27	2.675	0.102
High (n=62)	43	19		

### Baseline PLR and NLR levels were not related to outcomes

**Figure 3:**
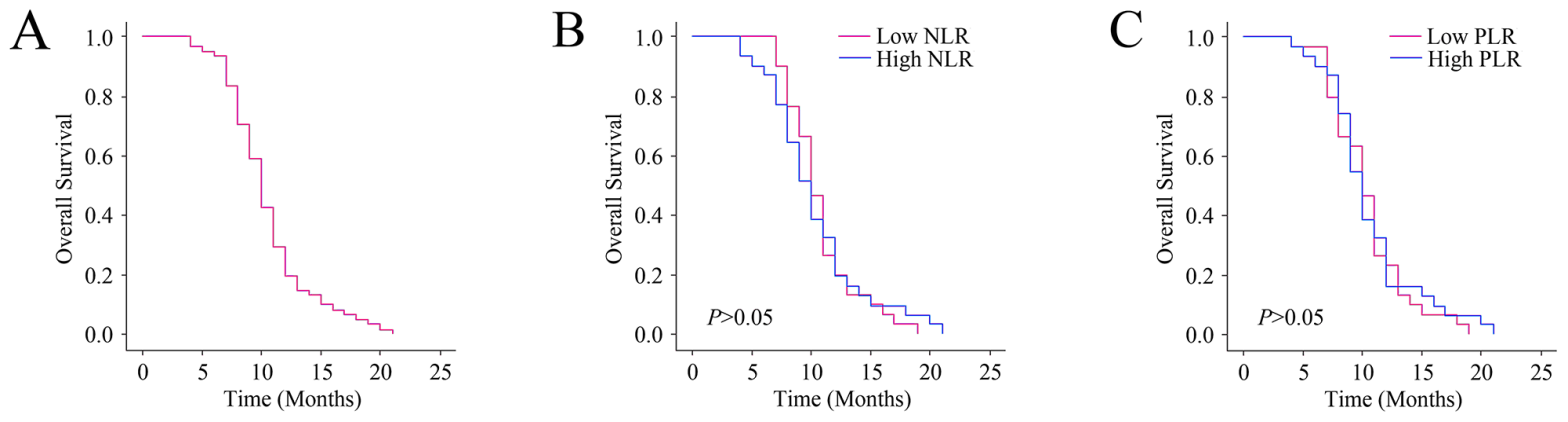
Relationship between baseline NLR and PLR levels and the outcomes **(A)** predicted probability of overall survival (OS). **(B)** The OS according to NLR. **(C)** The OS according to PLR.

The median OS of all patients was 10 months (9.73-10.27 months) (Figure 3A). Survivors were followed for 25 months. We used the Kaplan-Meier diagrams to evaluate the impact of the PLR and NLR states on OS (Figure 3B and 3C). The median OS was 10 months (9.52-10.48) for high NLR group, and 10 months (9.68-10.32) for the low NLR group respectively (*P*>0.05). The median OS of the high PLR group was 10 months (9.65-10.35), and the low PLR group was 10 months (9.65-10.35) (*P*>0.05). Therefore, baseline PLR and NLR levels were not correlated with outcomes.

### Changes in NLR level after chemotherapy were related to chemotherapeutic efficacy

To identify the relationship between changes of PLR or NLR levels and chemotherapeutic efficacy, blood samples were obtained and computed tomography (CT) scan assessments were performed at the same time subsequent to first-line chemotherapy. After chemotherapy, 42 patients with low baseline NLR levels retained (Table 4), while 18 patients changed to high NLR group. 46 patients with high baseline NLR levels reserved in this group. In contrast, 16 patients with high baseline NLR levels changed to low NLR group. Patients with low NLR levels after first-line chemotherapy (no matter remained or transferred to) had improved responses comparing with those retained or were transferred to high NLR group.

**Table 4:** Relationship between changes in NLR level and chemotherapeutic efficacy

Pre-chemotherapy	Post-chemotherapy	PR+SD (n=76)	PD (n=46)	χ2	*P* value
Low (n=60)	Low (n=42)	36	6	4.494	0.034
	High (n=18)	11	7		
High (n=62)	Low (n=16)	11	5	4.183	0.041
	High (n=46)	18	28		

After first-line chemotherapy, 30 patients were still reserved in low baseline PLR level group (Table 5), while 30 patients were transferred to high PLR group. Meanwhile, 34 patients in high baseline PLR level group maintained in this group, and 28 patients from this group changed to low PLR level group. Patients with low PLR levels after first-line chemotherapy (no matter remained or transferred to) did not exhibit improved responses comparing with those remained or transferred to high PLR level group (Table 5).

**Table 5:** Relationship between changes in PLR level and chemotherapeutic efficacy

Pre-chemotherapy	Post-chemotherapy	PR+SD (n=76)	PD (n=46)	χ2	*P* value
Low (n=60)	Low (n=30)	18	12	0.606	0.436
	High (n=30)	15	15		
High (n=62)	Low (n=28)	21	7	0.766	0.382
	High (n=34)	22	12		

Therefore, changes in NLR level, but not PLR level, after chemotherapy associated with chemotherapeutic efficacy.

### Changes in NLR level predicted outcomes

**Figure 4:**
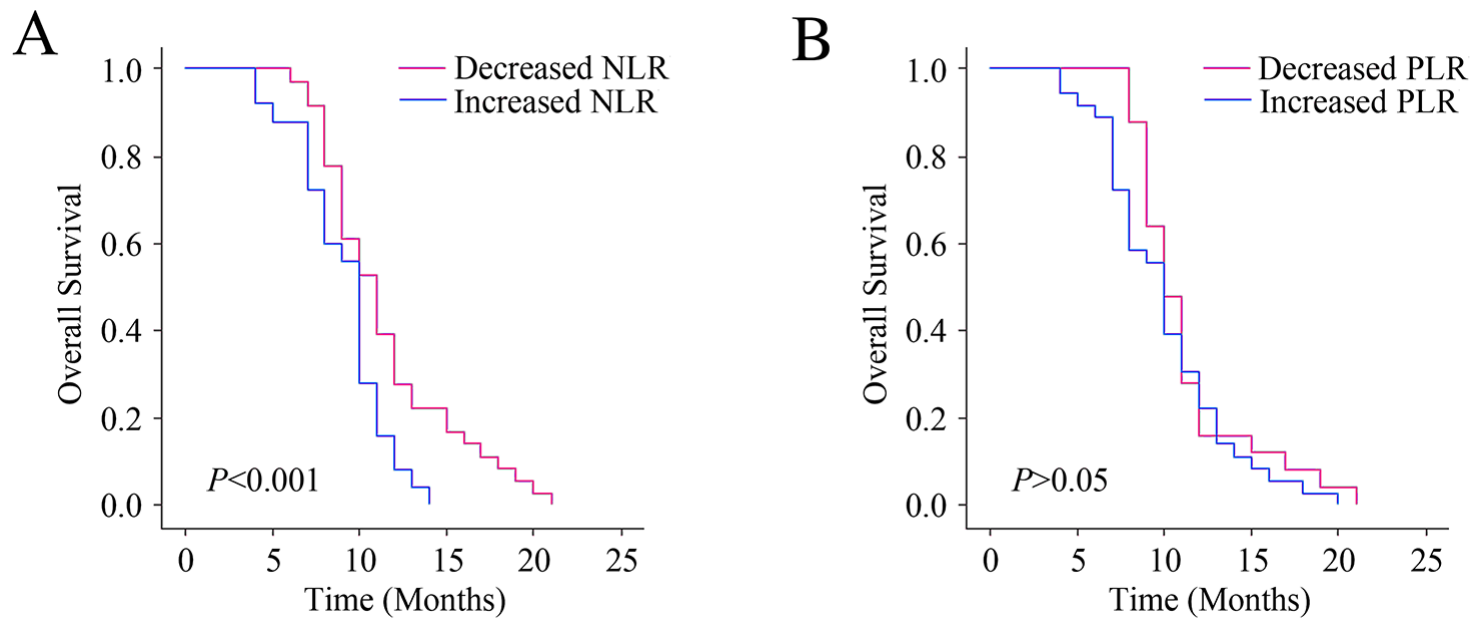
Relationship between changes in NLR and PLR levels after chemotherapy and the outcomes **(A)** The OS according to changes in NLR. **(B)** The OS according to changes in PLR.

The Kaplan-Meier diagrams were performed to evaluate the impact of changes in the PLR or NLR levels on OS (Figure 4). The median OS was 10 (9.77-10.23) months for patients with elevated NLR levels after first-line chemotherapy, comparing with 11 months (10.48-11.52) for NLR-decreased patients (*P*<0.001). The median OS of PLR-increased group was 10 months (9.65-10.35), and the PLR-decreased group was 10 months (9.61-10.39) (*P*>0.05). As a result, the survival rates of patients with elevated NLR levels after chemotherapy is reduced. But the changes in PLR level did not show prognostic value.

To evaluate the factors which were related to OS, univariate and multivariate analyses were further performed. By using the post-/pre-chemotherapy PLR or NLR ratios, patients were separated into two groups respectively. Post-/pre-chemotherapy ratios <1 represented that the values of NLR or PLR were decreased after chemotherapy, and ≥1 implied that PLR or NLR values were not decreased. As shown in Table 6, univariate and multivariate analysis confirmed that post/pre-chemotherapy NLR ratio is a prognostic factor affecting OS. Therefore, changes in NLR level, but not PLR level, after chemotherapy predicted outcomes.

**Table 6:** Univariate and multivariate analysis of risk factors for the overall survival

Risk factors	Overall survival (OS)			
	Univariate analysis		Multivariate analysis	
	OR (95% CI)	*P* value	OR (95% CI)	*P* value
**Gender** (Women or Men)	0.770 (0.528-1.124)	0.175	-	-
**Age** (>65 years or ≤65years)	0.773 (0.537-1.110)	0.163	-	-
**PLR** (high or low)	0.922 (0.641-1.325)	0.660	-	-
**NLR** (high or low)	1.007 (0.701-1.448)	0.969	-	-
**Post/pre-chemotherapy PLR ratio** (>1 or ≤1)	1.272 (0.881-1.837)	0.199	-	-
**Post/pre-chemotherapy NLR ratio** (>1 or ≤1)	1.902 (1.290-2.803)	0.001**	1.902 (1.290-2.803)	0.001**

## DISCUSSION

Inflammation, as a major feature of tumors [11], has been proved to be a crucial and essential process in the progression of malignancies [12] [13], including proliferation, angiogenesis, metastasis and chemotherapy-resistance [11]. Pancreatic cancer has a rather unique and complex microenvironment which is rich in inflammatory cytokines, growth factors and proteinases that are favorable for proliferation, invasion and metastasis of pancreatic cancer cells [14] [15]. Increased serum levels of inflammatory cytokines are also related to clinical features of pancreatic cancer, such as poor performance status, cachexia and OS [16, 17]. Therefore, cancer-associated inflammation is a key molecular feature in pancreatic cancer.

Systemic inflammation is considered to be able to predict poor outcomes among several types of malignances, including pancreatic cancer [18]. So far, lots of systemic inflammatory markers have been found to have predictive value for prognosis [19, 20]. For example, as a SIR marker, incorporating C-reactive protein (CRP), together with serum albumin, has a close relationship with outcomes in cancer patients [9]. Besides, serum lactate dehydrogenase (LDH) levels are also associated with the systemic inflammatory response and serves as a significant prognostic predictor in pancreatic cancer [21]. Systemic inflammation can also induce measurable neutrophilia and relative lymphocytopenia in the peripheral blood [19].

Neutrophils play an active role in both systemic and local inflammatory response. It is believed that up-regulation of neutrophils can reflect an aggressive feature of cancer cells since it is primarily stimulated by hematopoietic cytokines derived from tumor cells [22]. Neutrophils can produce arginase, nitric oxide (NO), reactive oxygen species (ROS), and inhibit the function of cytotoxic lymphocytes [23, 24]. In addition, preclinical researches have showed that neutrophils functioned as tumor-promoting factors via the signaling pathway triggered by transforming growth factor β (TGF-β) [25].

Lymphocytes, on the other hand, possess the anti-cancer activities through repressing tumor growth or metastasis [26]. Lymphocyte counts can reflect endogenous cancer resistance of the immune system [27, 28]. It has been proved that higher number of lymphocytes predicts better outcomes [27, 28]. Elevated numbers of lymphocytes might be related to a tumor shrink after neoadjuvant chemoradiotherapy in locally advanced rectal cancer [29].

By the combination of neutrophils and lymphocytes, high NLR level has been shown to be a promising indicator of poor prognosis for patients with resectable colorectal cancer [6]. Elevated NLR level is suggested as a marker for estimating benefits stage II colon cancer patients obtained from adjuvant treatment, and could identify risks of those patients [30]. Baseline NLR measurements and changes in NLR levels after therapy also have predictive values in outcomes of patients with unresectable gastric cancer [31]. Besides, NLR was also useful in determining prognosis in patients with non-small cell lung cancer cases [32]. Similar results have been found in patients bearing pancreatic cancer. Previous investigations have proved that elevated NLR level correlated with unfavorable prognosis in patients who received curative resection [33] [34] [35] or bypass surgery [36]. The pretreatment NLR could predict survival for patients with advanced pancreatic cancer who received systematic chemotherapy as well [37]. In our present study, we found the elevated NLR level in newly diagnosed pancreatic cancer patients than in healthy subjects. Moreover, patients whose NLR values increased after chemotherapy, predicted worse outcomes. Univariate and multivariate analysis also confirmed that post/pre-chemotherapy NLR ratio is a prognostic marker affecting OS in pancreatic cancer, suggesting the application of NLR not only possesses diagnostic value but also be able to provide prognostic information in pancreatic cancers.

Platelets also play a crucial role in the development of tumors [23]. Researches have proved that platelets can promote angiogenesis, extracellular matrix degradation, release of growth factors and adhesion molecules, all of which are the basic parts for the proliferation and metastasis of tumors. Thus platelets are able to accelerate cancer development [10]. In the blood stream, covering with platelets helps circulating tumor cells (CTCs) to overcome the pressure in the blood, involving the attack from immune system and physical factors [38]. Besides the effect of platelet on the natural course of cancer, number and function of platelets could also be influenced by cancer. Increasing numbers of studies suggested that the proinflammatory cytokines released by tumors, like interleukin-1 (IL-1), interleukin-3 (IL-3), and interleukin-6 (IL-6), can promote megakaryocyte proliferation, and lead to thrombocytosis gradually [10]. Therefore, an alliance of platelet and cancer provides a positive feedback on the progression of malignancies. In view of this, the platelet derived biomarkers present special potential.

By the combination of platelets and lymphocytes, PLR is thought to represent endogenous anticancer proinflammation and precoagulation in malignant tumors [9]. Besides, PLR is also assumed to be both a sensitive marker and a prognostic factor for breast cancers, ovarian cancers and colorectal cancers [39]. However, comparing with the value of NLR, many studies suggest that PLR levels are not qualified to be an independent prognostic factor for survival [10]. Our present findings indicated that, newly diagnosed pancreatic cancer patients have significantly higher PLR values comparing with the healthy controls, suggesting PLR could be a promising and easily available biomarker for the diagnosis of pancreatic cancer. It has been proved that cigarette smoke is a strong stimulator of pulmonary inflammation [40] [41]. In fact, elevated NLR and PLR levels have been observed in chronic obstructive pulmonary disease [42] [43] [44]. Therefore, the up-regulation of NLR and PLR could be correlated with both smoking history and cancer status, both of which are inflammation-related. Since both NLR and PLR are biomarkers reflecting systemic inflammation level, there is no surprise that patients with higher baseline NLR levels also possessed higher baseline PLR levels and vice versa. However, only NLR, but not PLR, associated with liver metastasis. Moreover, changes in PLR values after chemotherapy did not correlate with the response to treatment and overall survival, suggesting PLR is not as sensitive as NLR for the prognostic evaluation of pancreatic cancer, in consisting with a previous study [45].

Our study suggested that both NLR and PLR could be used in the diagnosis of pancreatic cancer. However, only NLR can be used to predict chemotherapy response and follow-up. In view of economic conditions in some areas of the world, this convenient, safe and lower cost biomarker may be beneficial for the patients with pancreatic cancer.

## MATERIALS AND METHODS

### Subjects and inclusion criteria

The study was conducted as a retrospective investigation of pancreatic cancer patients that had been referred to the First Affiliated Hospital of Soochow University (Jiangsu, China) between Jan 2007 and Mar 2015. Approval for the study was granted by the Medical Ethics Committees of the First Affiliated Hospital of Soochow University. Clinical and pathological records of all the patients participating in the study were reviewed periodically.

In total, 122 pancreatic cancer patients were recruited in this study. Patients had histologic or cytologic evidence of locally advanced or metastatic adenocarcinoma of the pancreas. Patient characteristics are detailed in Table 1. The mean age of the 122 patients was 65 years (range, 32–80 years), and 74 patients were male and 48 were female. The inclusion criteria were as follows: (a) those with histologically or cytologically confirmed recurrent or metastatic gastric cancer; (b) age more than 18 years; (c) Karnofsky performance status (KPS) score of≥70; (d) those with a predicted survival of ≥3 months; (e) either naive to anti-tumor treatment or the post-operative adjuvant chemotherapy was done at least six months after the last dose of chemotherapy; (f) in case of patients who were scheduled for radiotherapy on the target lesion, radiotherapy must have been finished for at least three months; (g) those with at least one measurable lesion (minimum 10mm×10mm on computed tomography (CT) or magnetic resonance imaging (MRI)); and (h) those who met the following laboratory criteria: white blood cells (WBC) ≥4.0×10^9^/L; absolute neutrophil count (ANC) ≥1.5×10^9^/L; platelet (PLT) ≥100×10^9^/L; Serum bilirubin ≤ upper limit of normal (ULN); alanine aminotransferase (ALT), aspartate aminotransferase (AST), and alkaline phosphatase (ALP) ≤ ULN×2.5 (if without liver metastasis) or ≤ ULN×5 (if with liver metastasis); urea nitrogen ≤ ULN×1.25; and creatinine ≤ ULN×1.25. The staging of cancer was made according to tumor–nodulus-metastases (TNM) classification and classified through the American Joint Committee on Cancer (AJCC) recommendations, 7th edition [46]. Patients were followed regularly for 60 months. The prognostic analyses were performed regarding progression-free survival (PFS) and overall survival (OS). 30 age–sexes matched healthy subjects were also included into this study.

### Blood samples

Peripheral venous blood (5-7 ml) was collected into a sterile ethylenediaminetetraacetic acid (EDTA) tube. All blood samples were fasted and obtained between 6:30 and 7:30 a.m. in order to standardize the known impact of circulating hormones (circadian rhythm) on the number and subtype distribution of the various white blood cell indices. Hematological parameters were analyzed within 30 min after collection using a hematology analyser (Sysmex XE-2100; Sysmex, Kobe, Japan). Neutrophil (10^3^/μl), lymphocyte (10^3^/μl) and platelet (10^3^/μl) counts and mean platelet volume (MPV) levels were recorded. NLR and PLR were calculated as the ratio of the neutrophils and platelets to lymphocytes, respectively. Mean value was used for NLR and PLR as normal distribution was absent. The patients were divided into two groups according to the mean value of NLR or PLR [(NLR low, <3.81 or NLR high, ≥3.81; and PLR low, <142.14 or PLR high, ≥142.14, respectively]. The post-/pre-operative ratios were defined as the rate of pre-operative PLR or NLR values and the corresponding ones obtained one month after operation.

### Chemotherapy and evaluation

In this study, most of the patients (98.4 %) underwent gemcitabine-based chemotherapy and a minority of the patients (1.6 %) underwent fluorine pyrimidines (fluorouracil, capecitabine, or S1 [tegafur, 5-chloro-2,4-dihydroxypyridine and oxonic acid]) treatment according to the newest edition of the National Comprehensive Cancer Network (NCCN) Guideline at the time. The regimen of gemcitabine alone was 1,000 mg/m^2^ (3 times per week followed by a 1-week rest for 6 cycles). Computed tomography (CT) scan was performed for the assessment of response every 2 months and evaluated according to the criteria of Response Evaluation Criteria in Solid Tumors (RECIST) 1.1 [47]. The responses to chemoradiotherapy including complete remission, regression, stable disease and disease progression.

### Follow-up

We recorded responses to chemoradiotherapy including complete remission, regression, stable disease, and disease progression, and overall survival (OS). After first line chemotherapy, disease progression after chemoradiotherapy was defined as lack of response to chemoradiotherapy. In contrast, stable disease, complete response or disease regression after chemoradiotherapy was defined as response to chemoradiotherapy. Patients were regularly followed for 25 months. Survival time was measured from the date of chemoradiotherapy until death or last clinical evaluation. The prognostic analyses were performed regarding overall survival (OS). OS was defined as the time from the diagnosed date to death from any cause.

### Statistical analysis

All statistical analyses were performed using SPSS 19.0 software (Chicago, USA). The associations between NLR or PLR levels and clinicopathologic features or chemotherapeutic efficacy were explored and assessed by the χ2 tests. For analysis of survival data, Kaplan–Meier curves were constructed, and statistical analysis was carried out using the log-rank test. Multivariate Cox regression was performed for each outcome parameter, using a backwards elimination technique to derive a potentially suitable set of predictors. All values of *P* < 0.05 were considered statistically significant.
